# Biological reflect of Adiponectin hormone in postpartum marital satisfaction and depression scores

**DOI:** 10.1186/s12884-020-03178-2

**Published:** 2020-09-11

**Authors:** Fattaneh Pahlavan, Anoshirvan Kazemnejad, Fatemeh Razavinia, Seyedeh Razieh Fazeli Daryasari, Najmeh Tahranian

**Affiliations:** 1 Department of Midwifery & Reproductive Health, Faculty of Medical Sciences, Tarbiat Modares University, Jalal Ale Ahmad Highway, P.O.Box: 14115-111, Tehran, Iran; 2 Department of Biostatistics, Faculty of Medical Sciences, Tarbiat Modares University, Tehran, Iran

**Keywords:** Adiponectin, Postpartum depression, Marital satisfaction, Delivery

## Abstract

**Background:**

There is a growing body of evidence regarding the association between Adiponectin and mental disorders. We aim to evaluate the association between serum level of Adiponectin hormone and postpartum depression and marital satisfaction scores.

**Methods:**

A prospective cohort study of 90 pregnant women was conducted in Mahdieh Hospital, Tehran, Iran. Blood samples were collected during the first 24 h after delivery. The serum Adiponectin concentration was measured with an Enzyme-Linked Immunosorbent Assay (ELISA) kit. The depression score was measured using a validated Iranian version of the Edinburgh Postnatal Depression Scale (EPDS) questionnaire at six weeks (6-weeks) and twelve weeks (12-weeks) after delivery. Using the Kansas questionnaire at twelve weeks (12-weeks) after delivery, the marital satisfaction score was measured. The measurements were compared between two groups, satisfied and dissatisfied mothers. *P*-values lower than 0.05 were considered significant.

**Results:**

The mean serum level of Adiponectin was significantly higher in the dissatisfied group. It was 10.9 ± 13.4 μg/ml and 15.2 ± 17.7 μg /ml in the satisfied and dissatisfied groups, respectively (*P* = 0.04).

The postpartum depression scores of 6- and 12-weeks after delivery were significantly higher in the dissatisfied group. At 6-weeks after delivery, the postpartum depression scores were 3.6 ± 3 and 8.7 ± 5.6 in satisfied and dissatisfied groups, respectively. Those were 2.7 ± 2.7 and 7.6 ± 5 at 12-weeks after delivery, respectively. There was a significant difference statistically (*P* <  0.001).

**Conclusion:**

Mothers in the dissatisfied group, experienced higher depression scores at 12-weeks postpartum while they had shown higher serum Adiponectin levels at the first 24 h after delivery.

## Background

Depression is the leading cause of disability in women worldwide affecting almost 18.4 and 19.2% of women during pregnancy and postpartum, respectively [1, 2]. Some mothers encounter with signs and manifestations of depression during the first week after delivery that are called Postpartum Depression (PPD) [3]. The symptoms include persistent crying, anxiety, fatigue, insomnia, discomfort, and irritability [4]. Although PPD can disturb the mother-baby relationship and can threaten the stability of the family, however, it is not often diagnosed and is not treated [5].

Recent studies discovered the role of the proliferation of hippocampal cells on the prevention of depression. This type of neurogenesis can be stimulated by hormones, such as Adiponectin [2]. Adiponectin is a 244 amino acid protein that is associated with multiple metabolic processes in the body [6–8]. Adiponectin is secreted into the bloodstream and in the range of 10–5 μg/ml, forms a total of 0.01% of total serum proteins [6]. Generally, the level of Adiponectin is the same during the first trimester of pregnancy and the non-pregnancy state. Adiponectin’s level reaches its maximum before mid-pregnancy and then decreases and continues to fall till 5 to 8 weeks postpartum [9].

Previous studies discovered the association between Adiponectin and mental disorders such as depression, prenatal anxiety, and PPD [2, 10, 11]. Rebelo et al., (2015) revealed the significant relationship between Adiponectin and anxiety during pregnancy [11]. In addition, Rebelo et al., (2016) showed that non-depressed mothers during pregnancy had higher levels of Adiponectin during pregnancy and the postpartum period. However, it was not significant, statistically [2].

Still, no study has analyzed the role of Adiponectin on postpartum marital satisfaction. In addition, the mode of delivery, normal vaginal delivery (NVD), and cesarean section (C-section) have not been considered as an important effective factor in prenatal depression. This study considered the mode of delivery along with the Body Mass Index (BMI) into different groups as confounder factors when analyzing depression symptoms. This way, this study tried to find new biomarkers to predict postpartum depression and marital dissatisfaction. Hence, we designed a study aim to evaluate the association between serum level of Adiponectin hormone (at the first 24 h after delivery) and postpartum depression and marital satisfaction scores.

## Method

### Study design and protocol

A prospective cohort study with three follow-up visits was conducted in Mahdieh Hospital, Tehran, Iran, between May and August 2016. A convenience sample of pregnant women was recruited, when the women accessed the service for the third-trimester prenatal care. To meet the eligibility, the women with ages ranged from 18 to 40 years old with normal Body Mass Index (BMI) before pregnancy were selected.

The inclusion criteria were singleton pregnancy, free of smoking or alcohol consumption, or other drug abuse and psychiatric health (If the pregnant woman had Edinburgh Depression Scale score > 10 in the third trimester, they would not enter the study). Mothers with chronic and systemic diseases such as immune disorders, heart and respiratory disease, diabetes, fatty liver, and thyroid disorders did not enter the study. Exclusion criteria were any lack of collaboration of individuals in follow up, unpleasant stressful events during pregnancy (e.g., divorce, obstetric complication, and dystocia), assisted vaginal delivery (e.g., using forceps and/or vacuums) and cesarean hysterectomy.

The final sample was comprised of 90 subjects to detect significant differences in the rate of satisfaction between groups, with a power of 90% and a two-tailed 5% significant level. Samples were divided into two groups, 45 subjects with C-section, and 45 subjects with NVD (without a history of C-section).

### Covariates assessment

The covariates in this study included maternal age (years), gravidity, parity (number of delivery), maternal education, job, number of alive children, BMI, and the number of previous abortions. Subjects were then classified into satisfied and dissatisfied groups. It worth mentioning that all C-section cases were spinal anesthesia. There was no anesthetic for NVD cases.

The information was gathered using a structured demographic questionnaire which was applied in the third trimester. The gestational age was calculated from the first day of the last menstrual period (LMP). The gestational age was calculated based on the first-trimester ultrasound when LMP was suspicious. The mother’s weight (kg), height, and BMI were measured using the standard equation and single investigator.

### Serum level of Adiponectin measurement

Blood samples were collected by a trained clinician after birth, within 24 h after the delivery. The samples were centrifuged (4000 rpm) at 4 °C for 10 min. The serum was then separated and frozen at − 70 °C according to the standard cold chain for further analyses. Finally, the serum Adiponectin concentration was measured with an Enzyme-Linked Immunosorbent Assay (ELISA) kits (Human Adiponectin ELISA, Zell BioGmbH, Ulm, Germany) with a sensitivity of 0.1 μg/ml with an intra-assay coefficient of variation (CV) of 6.3%.

### Depression symptoms and marital satisfactory scores

The symptoms of depression were measured for all subjects during the follow-up (i.e., 6- and12-weeks postpartum), using a validated Iranian version of the Edinburgh Postnatal Depression Scale (EPDS) questionnaire [12–14]. The scale was applied by a trained interviewer. Higher scores indicated greater depressive symptoms.

Then, the Kansas questionnaire was applied at 12-weeks after delivery to measure the marital satisfaction scores. The validity and reliability of the Kansas test were evaluated (Cronbach’s alpha coefficient 0.98) in Iran [15]. Scores higher than 17 indicated marital satisfaction.

### Statistical analyses

Descriptive tables, descriptors (descriptive statistics), and the results of the study (inferential statistics) were analyzed by SPSS (version 21, SPSS, Inc., Chicago, IL, USA) software and the *P*-value less than 0.05 was considered significant. The baseline characteristics of the participants were described as absolute (n) and relative (%) frequencies for the covariates. The mean and the standard deviation (SD) of serum Adiponectin were measured 24 h after delivery. The mean and the standard deviation (SD) of EPDS scores were described at 6- and 12-weeks postpartum. The mean and the standard deviation (SD) of satisfactory scores were measured at 12-weeks postpartum. These descriptive measurements were compared between the two satisfied and dissatisfied groups.

The normality of the quantitative data was tested applying the Kolmogorov-Smirnov test. Normal and non-normal data were compared applying t-test and Mann-Whitney-U test, respectively. Also, binary logistic regression was used to examine the associations between marital satisfaction and serum level of Adiponectin hormone (at the first 24 h after delivery) and postpartum depression scores with 95% confidence intervals.

## Results

Mothers between 18 and 40 years old who lived with their partners and had wanted pregnancy were selected for this study (*N* = 90). The satisfied and dissatisfied groups were separated. There was no difference in the baseline characteristics of groups including age, gravidity, parity, number of previous abortions, number of living children, gestational age, BMI, employment, education, and type of delivery (Table 1).

**Table 1:** Characteristics of study participants according to their postpartum marital satisfaction

Variables		Post-partum marital satisfaction		***P***-value^a^
		Satisfied (n = 45)	Dissatisfied (n = 45)	
**Continuous Variables, (Mean ± SD**				
Age (year)		28.3 ± 5.6	28.3 ± 5.4	**0.98**
Gravidity		2.3 ± 1.2	2.2 ± 1.2	**0.86**
Parity		0.8 ± 0.7	1.0 ± 1.1	**0.76**
Number of previous abortions		0.4 ± 0.8	0.22 ± 0.5	**0.21**
Number of living children		0.8 ± 0.7	1.0 ± 1.1	**0.67**
Gestational age at delivery		39.0 ± 0.8	39.2 ± 0.9	**0.20**
BMI (before pregnancy)		23.7 ± 3.5	23.1 ± 3.4	**0.37**
**Categorical Variables, (n (%))**				
Type of delivery	NVD	23 (51.1%)	22 (48.9%)	**0.83**
	C-section	22 (48.9%)	23 (51.1%)	
Employment	Employed	44 (97.8%)	45 (100.0%)	**0.31**
	Unemployed	1 (2.2%)	0	
Education	Elementary	8 (17.7%)	8 (17.7%)	**1.00**
	Middle school and Upper	37 (82.2%)	37 (82.2%)	
Sex of neonate	Female	19 (42.2%)	24 (53.3%)	**0.29**
	Male	26 (57.8%)	21 (46.7%)	

^a^Obtained from t-student/ Mann-Whitney-U test for continuous variables and Pearson’s χ2 test for Categorical variables

In general, the mean serum level of Adiponectin for all samples (*n* = 90) was 13 ± 15.8 μg/ml. Mean serum level of Adiponectin in satisfied and dissatisfied groups were 10.9 ± 13.4 μg/ml and 15.2 ± 17.7 μg/ml, respectively. Applying the Mann-Whitney-U test, there was a significant difference (*P* = 0.04) in the mean serum level of Adiponectin between satisfied and dissatisfied groups.

The postpartum depression scores at 6- and 12-weeks after delivery were 6.2 ± 5.1 and 5.1 ± 5.1, respectively for all subjects (*n* = 90). The postpartum depression scores of 6-weeks after delivery in satisfied and dissatisfied groups was 3.6 ± 3 and 8.7 ± 5.6, respectively. The postpartum depression scores of 12-weeks after delivery in satisfied and dissatisfied groups were 2.7 ± 2.7and 7.6 ± 5.8, respectively. Using the Mann-Whitney-U test, there was a significant difference (*P* <  0.001) between the satisfied and dissatisfied groups (Table 2).

**Table 2:** Comparison between Post-partum maternal depression and satisfaction scores and Adiponectin hormone levels according to postpartum satisfaction

Parameter	Number of samples	Post-partum marital satisfaction (Mean ± SD)		***P***-value^a^
		Satisfied	Dissatisfied	
**Serum levels of Adiponectin (μg/ml)**	Total (n = 90)	10.9 ± 13.4	15.2 ± 17.7	**0.04**^a^
	NVD (n = 45)	11.4 ± 11.2	14.9 ± 18.2	**0.54**
	C-section (*n* = 45)	10.38 ± 15.6	15.4 ± 17.7	**0.04**^a^
**Depression score (6-Weeks)**	Total (n = 90)	3.6 ± 3	8.7 ± 5.6	**< 0.001**^a^
	NVD (n = 45)	3.3 ± 2.6	8.7 ± 5	**< 0.001**^a^
	C-section (n = 45)	4.0 ± 3.5	8.7 ± 6.2	**0.01**^a^
**Depression score (12-Weeks)**	Total (n = 90)	2.7 ± 2.7	7.6 ± 5.8	**< 0.001**^a^
	NVD (n = 45)	3.3 ± 3.1	7.9 ± 5.7	**< 0.001**^a^
	C-section (n = 45)	2.0 ± 2.2	7.4 ± 6	**< 0.001**^a^
**Satisfaction score (12-Weeks)**	Total (n = 90)	18.3 ± 1	13.5 ± 2.2	**< 0.001**^a^
	NVD (n = 45)	18.4 ± 1.5	13.8 ± 2.4	**< 0.001**^a^
	C-section (*n* = 45)	18.8 ± 1.4	13.2 ± 2.1	**< 0.001**^a^

^a^Obtained from Mann-Whitney-U test

Binary logistic regression showed the odds ratio of dissatisfaction in individuals with higher serum levels of Adiponectin hormone was 1.01 (OR = 1.01(95%CI: 0.98, 1.04), *P* = 0.21)), and this result was not statistically significant. But it showed that the odds ratio of dissatisfaction in individuals with higher scores of depression of weeks 6 after delivery was 1.29 (OR = 1.29(95%CI: 1.14, 1.47), *P* <  0.001)). It was 1.28 (OR = 1.28(95%CI: 1.13, 1.44), P <  0.001)) in 12 weeks after delivery, which was statistically significant (Table 3).

**Table 3:** Predictors of postpartum marital satisfaction using the logistic regression model

Parameter	B	S.E.	p-value	Odds Ratio	95% CI (confidence interval) for Odds Ratio	
					Lower	Upper
**Serum levels of Adiponectin (μg/ml)**	0.01	0.015	**0.21**	**1.01**	0.98	1.04
**Constant**	−0.23	0.28	**0.39**	**0.78**		
**Depression score (6-Weeks)**	0.26	0.06	< 0.001^*^	**1.29**	1.14	1.47
**Constant**	−1.51	0.42	< 0.001^*^	**0.22**		
**Depression score (12-Weeks)**	0.24	0.06	< 0.001^*^	**1.28**	1.13	1.44
**Constant**	−1.16	0.35	< 0.001^*^	**0.31**		

Also considering BMI and mode of delivery (NVD and C/S) as other covariates, the adjusted odds ratio was measured (OR = 1.01(95%CI: 0.97, 1.04), *P* = 0.54)), and this result was not statistically significant (Table 4).

**Table 4:** Adjusted Odds Ratio for postpartum marital satisfaction using the logistic regression model

	B	S.E.	p-value	Odds Ratio	95% CI (confidence interval) for Odds Ratio	
					Lower	Upper
**Serum levels of Adiponectin (μg/ml)**	0.01	0.01	**0.54**	**1.01**	0.97	1.04
**Mode of delivery**	0.54	0.56	**0.34**	**1.71**	0.56	5.22
**BMI**	−0.11	0.07	**0.13**	**0.88**	0.76	1.03
**Depression score (6-Weeks)**	0.15	0.09	**0.10**	**1.17**	0.97	1.41
**Depression score (12-Weeks)**	0.15	0.09	**0.10**	**1.17**	0.97	1.41
**Constant**	0.76	1.72	**0.65**	**2.15**		

## Discussion

To the best of our knowledge, no study has compared the serum level of Adiponectin (at the first 24 h after delivery) and postpartum depression scores between satisfied and dissatisfied mothers. The main factors affect hormonal and behavioral state during pregnancy and postpartum are BMI, age, parity, gravidity, ethnicity, number of abortion, living child, gestational age, sex of neonate, smoking, history of postpartum depression, stressful signs of depression during pregnancy and childbirth, neonatal disorders, poor social support, low socioeconomic status, obstetric complications such as preeclampsia and stressors during pregnancy [5, 16]. In the present study, we omitted the confounding factors by matching the groups (Table 1) or considering them in inclusion or exclusion criteria.

The results of this study demonstrated that the serum level of Adiponectin (at the first 24 h after delivery) was significantly higher in the dissatisfied group compered to the satisfied group. In other words, mothers with lower satisfaction scores experienced higher depression scores at 12-weeks postpartum and had shown a higher serum level of Adiponectin at the first 24 h after delivery (Table 2). These results did not agree with the findings of a meta-analysis study of 506 depressed patients and 3714 controls. Studies have revealed that insufficient Adiponectin hormone associates with detected major depression [17]. It is proved that patients with depression had lower Adiponectin levels than healthy subjects [10].

Generally, Adiponectin applies its effect by expressing the receptors in the cerebral, cortex, hypothalamus, and hippocampus [18]. These hormonal secretions and expressions protect the neurons, motivate neurogenesis, and apply anti-depressant effects which can lead to a better mental function [19, 20].

In our study, the Adiponectin level (at the first 24 h after delivery) was significantly higher in the dissatisfied group that had higher postpartum depression scores at 12-weeks postpartum. A plausible explanation is that the increase of Adiponectin may be a hormonal compensatory mechanism of the body in the first stage of mental stress, inflammation, and depression. As a pathophysiologic clue, studies have shown that Adiponectin has an antidepressant activity mechanism in mice. Intracerebroventricular administration of Adiponectin has an antidepressant effect in mice. Adiponectin accelerates the growth of the hippocampal neural stem cells by activating the specific enzymes and proteins [20].

Jeong et al., (2012) stated that Adiponectin level increases in the patient with depression in the first stage (subsyndromal depression) which confirms the result of this study [21]. Moreover, another study was conducted by Yildiz et al., (2017) revealed that leptin and Adiponectin increase in the onset of postpartum depression. The results of this study agreed with the findings of our study. However, they just focused on the NVD group with no assessment of marital satisfaction scores [18].

On the other hand, Rebelo et al., (2016) found no correlation between the Adiponectin level and depression in pregnancy and at 30–45 days after delivery [2]. However, in their study, depressed mothers who had depressive disorder according to inclusion criteria were not excluded at first and the mode of delivery has not been considered.

In the current study, considering that depressed pregnant women were not entered the study, it can be claimed that after eliminating confounding factors and controlling the exacerbating factors, the result is more logical. So far, no such conclusion has been deduced from previous studies. It is the first study that has explored the relationship between the maternal serum concentration of Adiponectin and postpartum depression and marital satisfaction.

If Adiponectin and scores of PPD are considered as predictors of dissatisfaction, in binary logistic regression the odds ratio of them will be upper than one. As it is shown in Table 3, dissatisfaction is 1.29 and 1.28 fold higher in mothers with higher PPD scores of 6 and 12 weeks after delivery, respectively and it is significant (OR = 1.29 (95%CI: 1.14, 1.47), *P* <  0.001)) and (OR = 1.28(95%CI: 1.13, 1.44), P <  0.001)). Although the odds ratio of Adiponectin was upper than one, it was not significant (OR = 1.01(95%CI: 0.98, 1.04), *P* = 0.21).

One of the limitations in our study was the lack of hormonal assessment in each follow-up stage (6- and 12-Weeks). Further studies are required to firm evidence in favor of considering the Adiponectin as a new biomarker to predict and treat postpartum depression and marital satisfaction.

## Conclusion

The finding of this study revealed that dissatisfaction is significantly more in individuals with higher PPD scores of 6 and 12 weeks after delivery. Mothers in the dissatisfied group at 12-weeks postpartum experienced higher depression scores, and significantly had shown higher serum Adiponectin at the first 24 h after delivery. Increase of Adiponectin may be a hormonal compensatory mechanism of the mother, in the first stage of mental stress, inflammation, and depression. Considering this, Adiponectin can be a predictive biomarker for early detection of depression that helps to prevent postpartum depression and marital dissatisfaction.

## Data Availability

Data supporting our findings can be sent upon request.

## Abbreviations

ELISA: Enzyme-Linked Immunosorbent Assay
EPDS: Edinburgh Postnatal Depression Scale
PPD: Postpartum Depression
NVD: normal vaginal delivery
C-section: Caesarian section
BMI: Body Mass Index
